# Danggui Buxue Decoction attenuates 4-(methylnitrosamino)-1-(3-pyridyl)-1-butanone—induced lung cancer growth in A/J mice by suppressing HIF-1α/VEGF-mediated angiogenesis

**DOI:** 10.3389/fmed.2025.1687685

**Published:** 2025-10-22

**Authors:** Wu Chen, Ying Guo, Huan Liu, Yushan Zhang, Sanyin Zhang, Zhilong Liu

**Affiliations:** ^1^Chongqing Hospital of The First Affiliated Hospital of Guangzhou University of Chinese Medicine (Chongqing Beibei Hospital of Traditional Chinese Medicine), Chongqing, China; ^2^Innovative Institute of Chinese Medicine and Pharmacy/Institute of Interdisciplinary Studies, Chengdu University of Traditional Chinese Medicine, Chengdu, China; ^3^Taian City Central Hospital, Taian, China; ^4^Outpatient Department of the 38th Ex-Cadre Sanatorium, Beijing, China; ^5^State Key Laboratory of Southwestern Chinese Medicine Resources, Innovative Institute of Chinese Medicine and Pharmacy/Academy for Interdisciplinary, Chengdu University of Traditional Chinese Medicine, Chengdu, China

**Keywords:** lung cancer, Danggui Buxue Decoction, angiogenesis, micro CT, vascular endothelial growth factor

## Abstract

**Background:**

Lung cancer (LC) persists as a leading cause of global cancer-related mortality. Pathological angiogenesis constitutes a critical mechanism in LC progression, facilitating neovascularization that supplies oxygen and nutrients to support tumor growth. Despite this, current anti-angiogenic therapies face significant clinical limitations. Danggui Buxue Decoction (DBD), a traditional Chinese herbal formula used to tonify Qi and activate blood circulation, exhibits clinical potential in delaying LC progression; however, its precise mechanistic basis remains incompletely defined. This study aimed to evaluate the inhibitory effects of DBD aqueous extract on lung tumors in 4-(methylnitrosamino)-1-(3-pyridyl)-1-butanone (NNK)-induced A/J mice and elucidate whether its therapeutic mechanism involves suppression of hypoxia-inducible factor-1α (HIF-1α)/vascular endothelial growth factor-A (VEGF)-mediated angiogenesis.

**Methods:**

Potential therapeutic targets of DBD against LC were identified through database mining (OMIM, TTD, GeneCards) using topological analysis. A/J mice received intraperitoneal injections of NNK (100 mg/kg) to induce lung tumors. From week 10, DBD aqueous extract (10 g/kg/day) was administered via oral gavage. Lung tumor progression and systemic parameters were assessed at weeks 10 and 20 using small-animal computed tomography (CT), enzyme-linked immunosorbent assay (ELISA), Doppler ultrasound, pulmonary function tests, and complete blood counts (CBCs). At week 20, mice were anesthetized with 3% isoflurane and sacrificed by cervical dislocation. Lung tissues were harvested for histopathological evaluation (H&E), immunohistochemistry (CD31), and immunofluorescence (HIF-1α/VEGF) to quantify microvessel density and hypoxia/angiogenesis markers.

**Results:**

Network pharmacology identified TP53, AKT1, MYC, and VEGF as core therapeutic targets of DBD. *In vivo*, small-animal CT detected pulmonary opacities at week 10, concomitant with elevated pulmonary artery flow, increased airway resistance, and heightened circulating levels of TNF-α, VEGF, white blood cells (WBC), and neutrophils. By week 20, progressive multifocal opacities emerged alongside reduced pulmonary artery flow, impaired lung function, elevated TNF-α/VEGF, and decreased WBC, lymphocytes, and platelets. Compared to untreated controls, 10-week DBD treatment significantly suppressed lung tumor growth, reduced lesion microvessel density, downregulated HIF-1α and VEGF expression, and ameliorated hematological dysregulation.

**Conclusion:**

Our findings indicate that angiogenesis serves as a core mechanism driving NNK-induced lung tumorigenesis in mice. DBD attenuates tumor growth primarily by inhibiting HIF-1α/VEGF-mediated angiogenesis, with complementary contributions from restored immune homeostasis and ameliorated hypoxia.

## Graphical abstract

**Figure:**
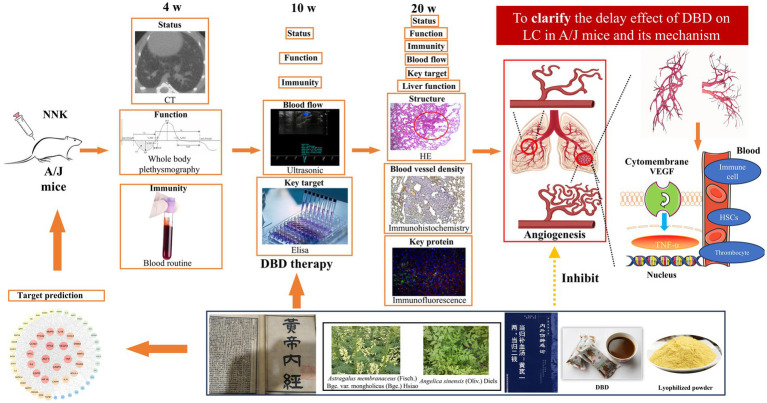
This figure was drawn by using Figdraw.

## Introduction

According to the American Cancer Society, lung cancer (LC) is projected to remain the most prevalent cancer worldwide in 2024, accounting for approximately 12.4% of all new cancer cases (1). The rising incidence of LC has been driven by environmental exposures, an aging global population, and the persistent prevalence of tobacco use. Furthermore, data indicates that the number of LC patients and mortality rates vary by gender and region, which means that standardized treatment regimens cannot meet the needs of patients (2).

Vascular invasion represents a hallmark of LC progression. Imaging studies consistently reveal elevated blood vessel density surrounding tumors or neovascular infiltration into tumor masses, both indicative of active angiogenesis. This process functions as an adaptive mechanism through which tumors establish alternative circulatory pathways to meet increasing metabolic demands when existing vasculature becomes insufficient (3). However, these newly formed vascular networks accelerate LC growth. Hypoxia serves as a central driver of this phenomenon. Under low oxygen conditions Hypoxia stabilizes hypoxia-inducible factor-1 alpha (HIF-1α) by inhibiting its ubiquitination. Stabilized HIF-1α subsequently upregulates pro-angiogenic mediators, particularly vascular endothelial growth factor (VEGF), which plays a pivotal role in pathological angiogenesis (4). Dysregulated VEGF expression promotes the formation of structurally abnormal, hyperpermeable, and edematous blood vessels. This aberrant vasculature further exacerbates tissue hypoxia and promotes vascular inflammation (5). Collectively, the HIF-1α/VEGF signaling axis constitutes a central regulatory pathway in LC angiogenesis and represents a key target for therapeutic intervention.

Anti-angiogenic strategies targeting tumor vascularization have been investigated for decades (6). While FDA-approved VEGF inhibitors, such as bevacizumab, demonstrate efficacy in advanced LC, their clinical utility is constrained by several inherent limitations: (1) Development of resistance mediated by vascular mimicry or hypoxia adaptation (7). (2) Significant adverse events, including pulmonary hypertension (affecting >15% of patients) and gastrointestinal perforation (8). (3) Inability to counteract tumor-induced immunosuppression (9–11). These limitations highlight the critical need for novel multi-target and anti-angiogenic agents with improved safety profiles and broader mechanisms of action.

**Figure 1:**
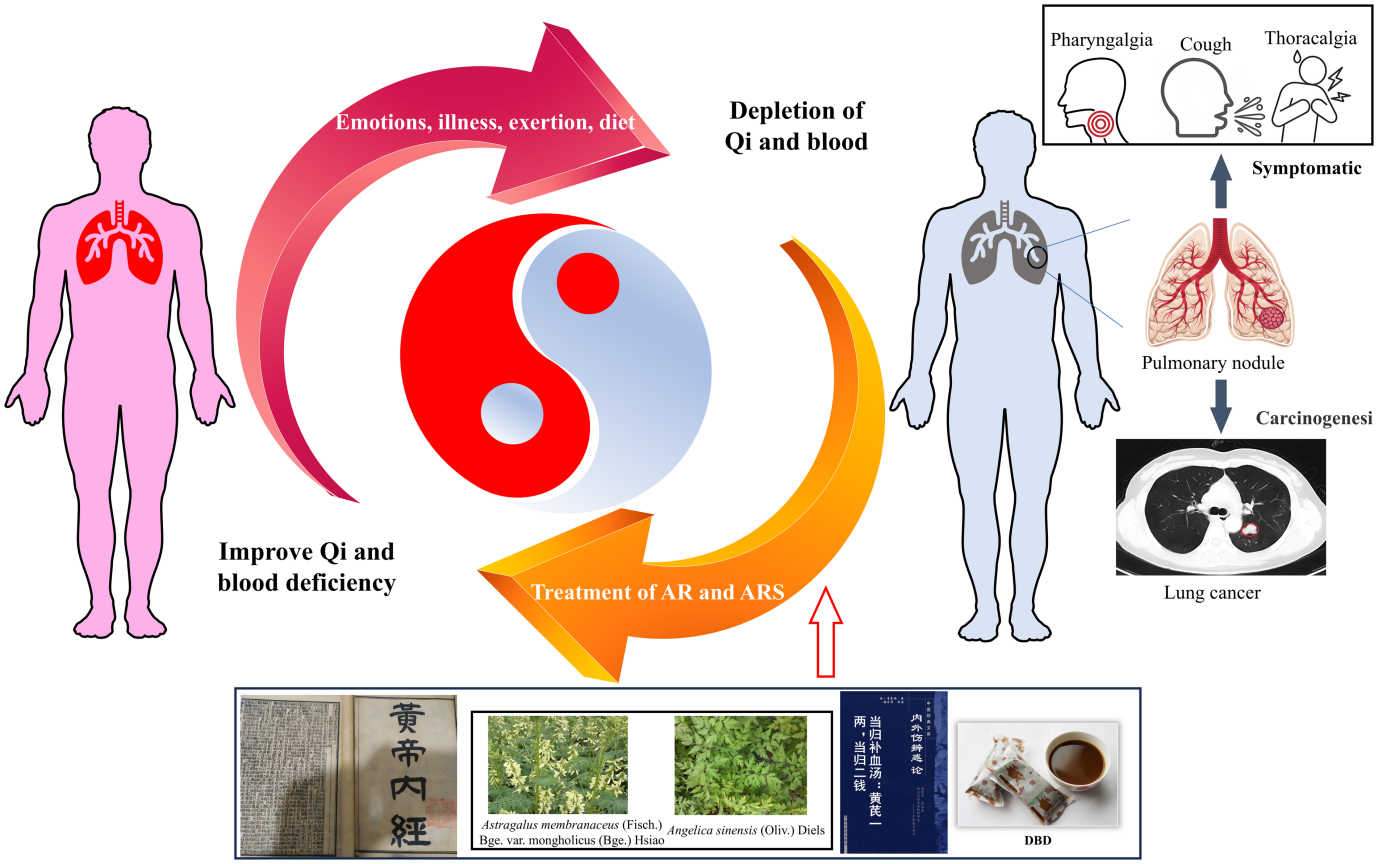
Illustrates the TCM perspective on the formation, progression, and carcinogenesis of LC. The development and carcinogenesis of LC are fundamentally influenced by the state of Qi and blood. A deficiency in Qi and blood contributes to the formation of LC, leading to symptoms such as sore throat, cough, and chest tightness. As the deficiency persists, LC grows in size, further aggravating the condition. DBD has been shown to effectively supplement Qi and blood, thereby delaying LC progression and mitigating associated symptoms. This figure was drawn by using Figdraw.

Danggui Buxue Decoction (DBD), a classical TCM formulation comprising *Astragalus membranaceus* (Fisch.) Bge. var. mongholicus (Bge.) Hsiao (Huang Qi, AR) and *Angelica sinensis* (Oliv.) Diels (Dang Gui, ARS), has been historically employed for centuries to tonify Qi and nourish blood in pulmonary conditions. Originally documented in Gao Li′s *Neiwaishang Bianhuo Lun* (Song Dynasty, 1247 A.D.), DBD constitutes a cornerstone prescription in TCM-based LC management. Within the TCM paradigm, LC pathogenesis (“Feiji”) is attributed to underlying Qi and blood deficiency compounded by external pathogenic invasion (12). Qi and blood deficiency can both impair the body’s ability to defend against external stimuli and hinder the elimination of pathological products. Therefore, TCM emphasizes the use of formulations based on DBD to tonify Qi and blood, thereby delaying the accumulation and growth of “Feiji.” Both the “*Guidelines for Integrated Traditional Chinese and Western Medicine Diagnosis and Treatment of Non-Small Cell Lung Cancer 2024*^1^” and the “*Expert Consensus on TCM Early Lung Cancer Screening and Prevention (2022)*” (Number PREPARE-2022CN742) recommend the use of prescriptions based on DBD for the treatment of LC patients (such as Buzhong Yiqi Decoction, Guipi Decoction, and Bazhen Decoction). Contemporary clinical studies indicate DBD modulates immune function, attenuates pulmonary inflammation, and inhibits tumor proliferation (13–15). Our prior research further established that DBD promotes hematopoiesis, regulates vascular tone, enhances tissue perfusion, and suppresses lung cancer cell growth (16–18). Nevertheless, whether DBD exerts its antitumor effects via suppression of HIF-1α/VEGF-mediated angiogenesis remains unclear. The TCM principle of employing Qi and blood supplementation for LC management is schematically represented in Figure 1.

Building upon clinical evidence and our previous findings, the current study utilized network pharmacology to predict DBD’s therapeutic targets against LC To experimentally validate these predictions *in vivo*, we established an orthotopic lung cancer model in A/J mice using the tobacco-specific carcinogen 4-(methylnitrosamino)-1-(3-pyridyl)-1-butanone (NNK). Following confirmation of tumor establishment, animals received DBD treatment. Tumor progression was subsequently assessed using multimodal evaluation techniques. This investigation sought to determine whether DBD’s therapeutic efficacy in delaying LC progression is mediated through inhibition of HIF-1α/VEGF expression and consequent suppression of angiogenesis.

## Materials and methods

### Experiment materials and animal modeling

Fifteen female A/J mice, weighing 18–20 g, were purchased from GemPharmatech (Nanjing, Jiangsu, China) and housed under standardized conditions, including a temperature of 25 ± 1 °C, air humidity of 50 ± 5%, and a 12-h light/dark cycle (07:00–19:00 light phase). Solid chow and tap water were provided with ad libitum. Following one-week acclimatization, mice were randomly divided into three groups (*n* = 5/group): model group (M), DBD treatment group (T), and blank control group (B). Groups T and M received a single intraperitoneal injection of 100 mg/kg 4-(methylnitrosamino)-1-(3-pyridyl)-1-butanone (NNK), while the B group received an equal volume of saline solution. Starting from the 10-week time point, the T group was orally administered 10 g/kg DBD (19). Groups M and B received equivalent volumes of distilled water. Body weights were recorded weekly. At the 20th week, the mice were anesthetized with 3% isoflurane and were sacrificed by neck removal. All experimental procedures adhered to the guidelines approved by the Management Committee of Chengdu University of TCM, Chengdu, Sichuan, China (Record No. 2024035).

### Ingredient targets in DBD and LC-associated targets

The chemical components of DBD (composed of AR and ARS) were retrieved from the Traditional Chinese Medicine System Pharmacology Database and Analysis Platform (TCMSP, https://old.tcmsp-e.com/tcmsp.php). The screening criteria were set as oral bioavailability >30% and drug-likeness >0.18. Compounds meeting these criteria were considered potential active ingredients and included in the database. All target proteins were submitted to the UniProt database^2^ for standardization, converted to the official gene symbols of *Homo sapiens*, and deduplicated to form the “Danggui Buxue Decoction-Compound-Target” database.

Using “Lung Cancer” as the keyword, a search was conducted in GeneCards,^3^ the Online Mendelian Inheritance in Man (OMIM, https://www.omim.org/), and the Therapeutic Target Database (TDD, http://db.idrblab.net/ttd/). After integrating and deduplicating the target proteins collected from each database, a comprehensive lung cancer-related target database was established.

Venny software was used to perform intersection analysis between the two aforementioned databases. The overlapping genes identified were defined as potential targets for DBD in the treatment of LC. To explore the interactions between these targets, the intersecting targets were uploaded to the STRING database (v12.0, https://string-db.org/) to construct a protein–protein interaction (PPI) network. The species was set as *Homo sapiens*, with a minimum confidence score of 0.400. Subsequently, the network data were imported into Cytoscape software (v3.9.1) for visualization, and the CytoNCA plugin was used to analyze the topological parameters of the network. The degree of nodes was calculated to identify key hub genes (20).

### Herbs and preparation of lyophilized powder

AR and ASR were obtained from the Affiliated Hospital of Chengdu University of TCM and authenticated by Professor Sanyin Zhang. The medicinal materials met the inclusion standards specified in the 2020 edition of the Chinese Pharmacopoeia.

To ensure consistency in drug quality, strict quality control was maintained during the preparation of lyophilized powder. Initially, AR and ASR were pulverized using grinder (RS-FS1401, Royalstar, China). The two herbs were then accurately weighed at a ratio of 5:1 (AR:ARS). The mixture was boiled with distilled water (10 × volume) at 100 °C for 1 h and centrifuged at 5,000 rpm for 10 min to obtain the supernatant. The herbal residue was then subjected to a second extraction with distilled water (10 × volume) under the same conditions. The combined supernatants were frozen at −80 °C overnight and lyophilized using a freeze dryer (Eyel4 Model, Tokyo Physicochemical, Japan). Before use, the lyophilized powder was dissolved in distilled water at a concentration of 1 g/mL, centrifuged at 5,000 rpm for 10 min, and filtered through a 0.22 μm microporous membrane (16).

### Micro-CT imaging and image analysis

Commencing from the initial NNK injection, pulmonary nodule progression was assessed via micro-CT scans at 4-, 10-, and 20-weeks post-injection (*n* = 5 mice/group/timepoint). Mice were anesthetized with 3% isoflurane and positioned in the prone position on the scanner bed (Quantum GX2, PerkinElmer, United States) with full thoracic exposure. Scan parameters: (i) X-ray source: Cu 0.06 + Al 0.5 filter; (ii) Voltage: 70 kV; (iii) Current: 80 μA; (iv) Rotation: 360°; (v) Field of view: 36 mm × 36 mm; (vi) Acquisition time: 4 min.

Reconstructed datasets were processed in 3D Slicer (version 5.6.2). Lesion regions were segmented using the Segment Editor module. Radiomic features were extracted via the Radiomics extension, while lesion volume and maximum diameter were quantified using the Segment Statistics tool (18, 19). Quantitative data were exported to SPSS 22.0 for statistical analysis and GraphPad Prism 8.0 for visualization.

### Histopathological analysis by H&E staining

On day 140 (20 weeks) post-initiation, mice were anesthetized with 3% isoflurane and sacrificed by cervical dislocation. Lung tissues from all groups (*n* = 5/group) were harvested and immersion-fixed in 4% paraformaldehyde (PFA) at 4 °C for 24 h. Following fixation, tissues underwent sequential processing: (i) Dehydration: Ethanol gradient (75%–85%–90%–95%–100%; 1 h/step); (ii) Clearing: Xylene immersion (2 × 1 h); (iii) Embedding: Paraffin infiltration at 60 °C (3 × 1 h) followed by embedding in paraffin blocks. Serial sections (4-μm thickness) were mounted on slides, deparaffinized in xylene (2 × 5 min), and rehydrated through graded ethanol (100% to 95%; 5 min/step). H&E staining was performed according to the previous protocol (21).

### Immunohistochemistry

Lung tissues were fixed in 4% paraformaldehyde, paraffin-embedded, and subjected to antigen retrieval. Subsequently, sections were incubated with 3% hydrogen peroxide in the dark for 15 min to block endogenous peroxidase activity. After a 60-min blocking step with 3% BSA, sections were incubated overnight at 4 °C with a CD31 primary antibody (Abcam, 1:50). After washing with PBS, sections were incubated with a fluorescent secondary antibody (Proteintech Group, Inc.) and subjected to ethanol gradient dehydration, clearing in xylene, and sealing with neutral gum. Images were captured under an inverted microscope (22). Different samples were detected using Image-J software (National Institutes of Health 1.8.0_112) to determine the positive area of CD31 and the gray value of HIF-1α/VEGF.

### Lung function measurement

Lung function was assessed at 4-week, 10-week, and 20-week time points using a whole-body plethysmography system (WBP) (23). The WBP system must be calibrated by injecting a defined volume of gas into the closed chamber. This process enables the calibration of the flow rate and pressure. Following calibration, the mice were placed in a scanning box and permitted to move freely for a period of 6 min. The data were recorded at 3-s intervals using the fine point setting. Following the conclusion of the experiment, the data was exported and retained (24).

### Lung artery blood flow measurement

Pulmonary artery blood flow was measured at the 10-week and 20-week time points using a small animal echocardiography system (VisualSonics Vevo 3100, Canada). Mice were anesthetized with isoflurane, positioned on a heating pad, and had chest hair removed. Using a 40 MHz pulsed Doppler ultrasound probe, pulmonary artery blood flow velocity was measured from the long-axis view of the sternum (25).

### The examination of complete blood count

Blood samples were collected via submaxillary artery puncture into EDTA-containing tubes. Blood was analyzed using a veterinary hematology analyzer (BC-2800Vet, Mindray, China) (26).

### Identification of enzyme-linked immunosorbent assay

Blood samples were collected at the 10-week and 20-week time points and centrifuged at 4 °C, 3,500 rpm to separate serum. Serum VEGF and tumor necrosis factor-α (TNF-α) levels were quantified using an enzyme-linked immunosorbent assay (ELISA) kit (Multiskan FC, Thermo Scientific, United States) at 450 nm absorbance, according to the manufacturer’s instructions.

### Liver function tests

At the 20-week time point, blood samples were collected and centrifuged to obtain plasma. Alanine transaminase (ALT) and aspartate transaminase (AST) levels were determined using an automatic biochemical analyzer with the following settings: 37 °C, 340 nm wavelength, 60-s delay, and 120-s detection time, following the kit protocol.

### Statistics analysis

Statistical analyses were performed using SPSS 23 (IBM, United States) and GraphPad Prism 8 (GraphPad, United States). Differences between two groups were analyzed using Student’s *t*-test, while one-way ANOVA was used for comparisons among multiple groups. Data were expressed as mean ± SEM, and statistical significance was set at *p* < 0.05.

## Results

### Network pharmacology identifies VEGFA as a core target for DBD in inhibiting lung cancer growth

**Figure 2:**
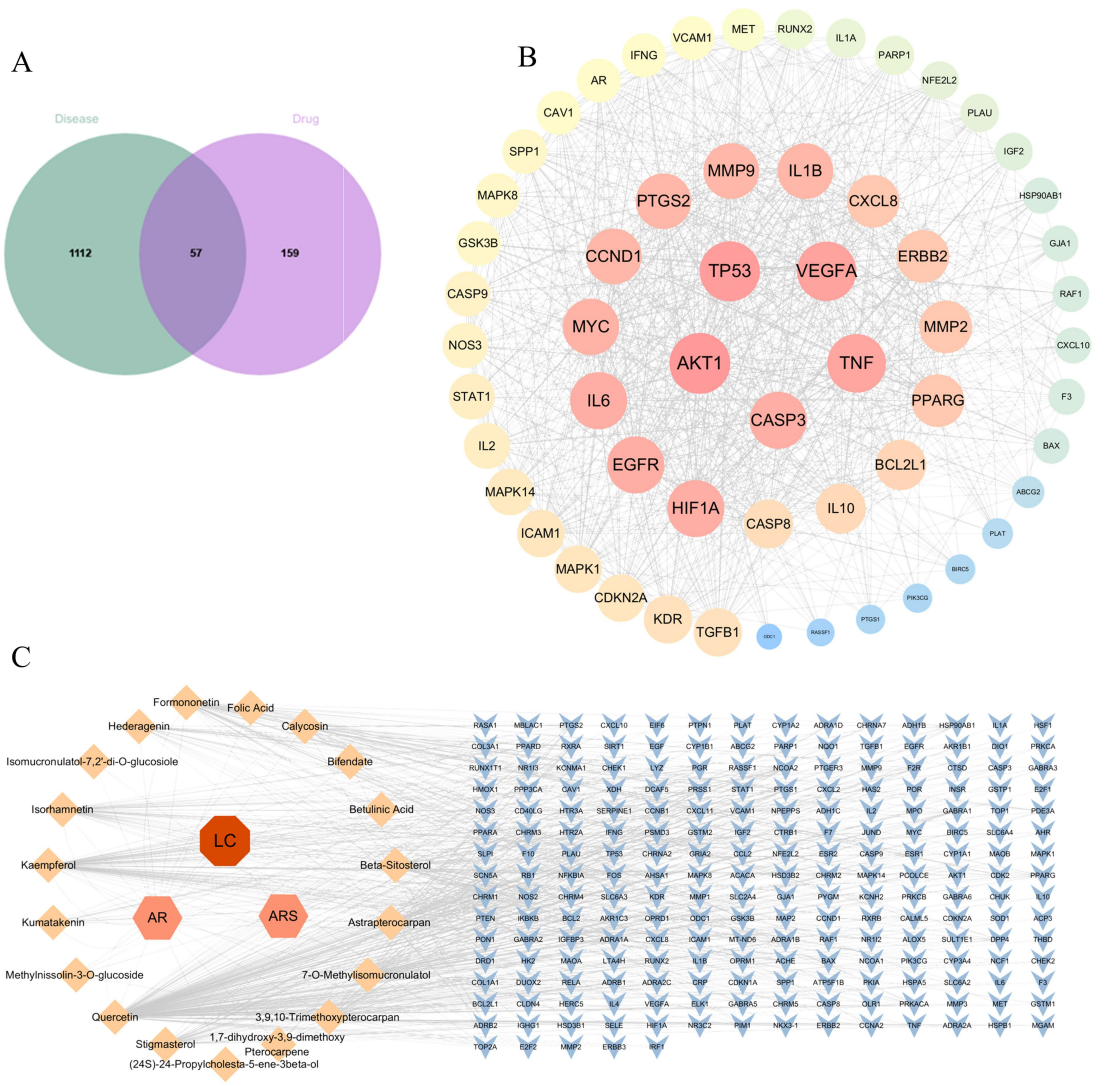
Network pharmacology analysis for DBD on LC. **(A)** Venn diagram showing the intersection between DBD-related targets (drug) and LC-related targets (disease), identifying 57 potential therapeutic targets. **(B)** Protein–protein interaction (PPI) network of the 57 potential targets. Node size and color intensity are proportional to the degree centrality, highlighting key hub genes such as AKT1, YP53 and VEGFA. **(C)** The “Component-Target” network illustrating the interactions between the active components of DBD (orange nodes) and their corresponding protein targets (blue nodes).

Database screening yielded 216 putative targets of DBD. Concurrently, 1,169 disease-related targets associated with LC were collated. Intersection analysis of these target sets identified 57 potential therapeutic targets for DBD in LC treatment. Subsequent topological analysis of the protein–protein interaction (PPI) network using Cytoscape revealed key hub genes based on degree centrality. The top five hub genes were AKT1, TP53, VEGFA, TNF, and CASP3. Notably, VEGFA emerged as the core hub gene, suggesting that the anti-angiogenesis effect mediated by VEGFA inhibition represents a central mechanism of DBD action against LC. These results are summarized in Figure 2.

### Micro-CT detection of lung lesions in mouse model

**Figure 3:**
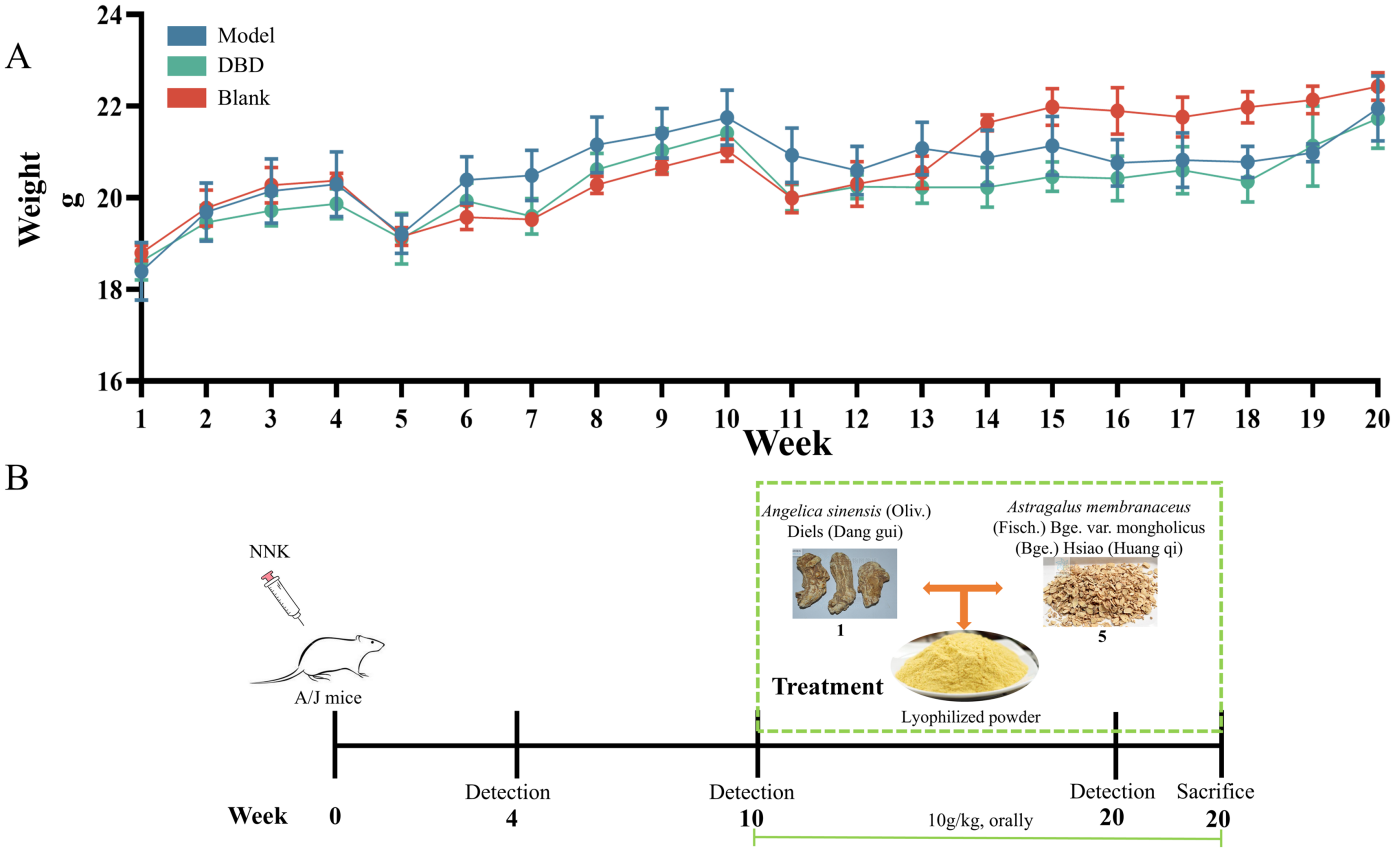
Effect of DBD treatment on the body weight of NNK-treated A/J mice and the experimental design. **(A)** Demonstrates that neither NNK treatment nor DBD treatment had a significant impact on the body weight of the experimental mice. **(B)** Provides a schematic representation of the model construction and treatment process. This figure was drawn by using Figdraw.

To evaluate the potential of DBD in delaying LC progression, an NNK-induced LC model was established in A/J mice. As shown in Figure 3A, neither NNK administration nor DBD treatment resulted in significant changes in mouse body weight throughout the study period. The detailed experimental timeline and DBD dosing regimen are outlined in Figure 3B.

**Figure 4:**
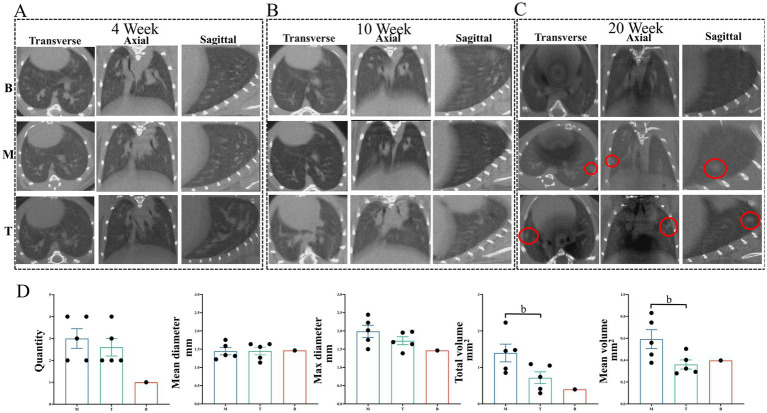
Imaging alterations in mouse lung tissue. **(A,B)** Show lung tissue morphology in mice observed using micro-CT at the 4-week and 10-week marks, respectively. **(C)** Illustrates lung morphology across the three experimental groups at the 20-week mark. The red circles highlight the LC foci from three different perspectives. **(D)** Presents quantitative data on the total number, mean diameter, maximum diameter, total volume, and mean volume of LC across the three groups at the 20-week mark. The red circles indicate the LC locations in the CT images. Statistical significance is denoted by b, indicating a significant difference between the model group and the DBD group (*p* < 0.05). M represents the model group, T represents the DBD group, and B represents the blank group. Data are expressed as mean ± SEM (*n* = 5).

Longitudinal micro-CT imaging was performed at 4, 10, and 20 weeks post-initiation to monitor LC development (19). At 4-week, no significant lung shadow foci were observed in any experimental group (Figure 4A). By 10-week, small shadow foci became detectable in some mice. Furthermore, mice in the model group (M) and the DBD treatment group (T) exhibited increased lung parenchymal density compared to the blank control group (B) (Figure 4B). At 20-week, group B lungs displayed normal texture, whereas group M lungs presented with solid shadow foci, disordered pulmonary architecture, and blurred lesion boundaries. In contrast, DBD treatment significantly these pathological changes, resulting in improved lung texture and a demonstrable delay in LC progression (Figure 4C). Quantitative analysis of the CT images revealed that while DBD treatment did not significantly reduce the total number of lesions, mean lesion diameter, or maximum lesion diameter (*p* > 0.05), it did induce a statistically significant reduction in both the average lesion volume and the total lesion volume (Figure 4D).

### Histopathological assessment of lung tissue by H&E staining

**Figure 5:**
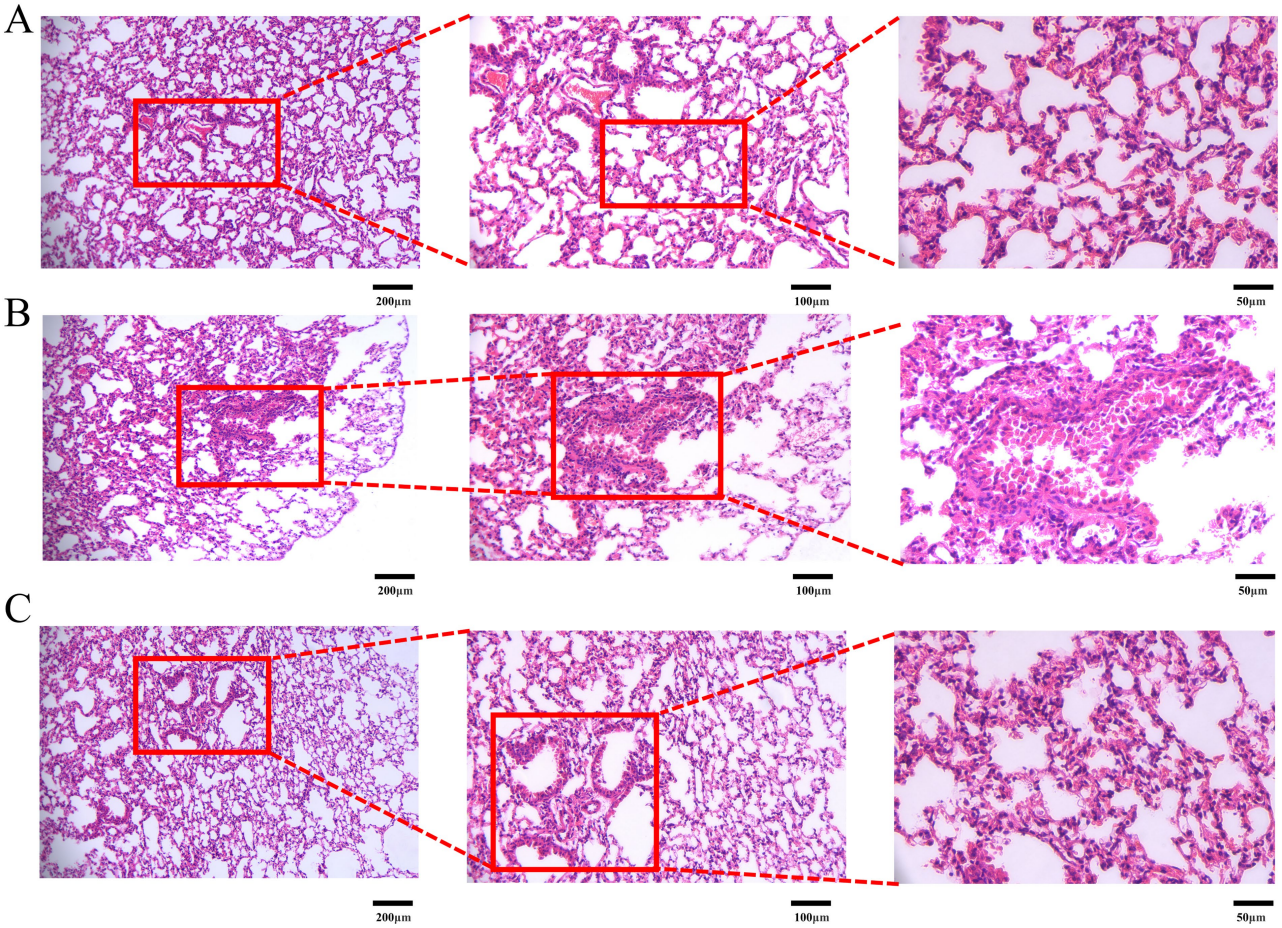
H&E staining of mouse lung tissue. **(A)** Represents the blank group. **(B)** Represents the model group. **(C)** Represents the treatment group. Images are presented at magnifications of 10×, 20×, and 40× from left to right, respectively. Data are based on observations from *n* = 5 mice per group.

Given the close association of LC growth with local inflammation and angiogenesis, H&E staining was employed to evaluate LC-induced lung injury and the therapeutic efficacy of DBD. As shown in Figure 5, a 10-week course of DBD treatment effectively preserved lung tissue structural integrity and mitigated the development of edema, inflammatory infiltration, and angiogenesis compared to the model group (M). Histopathological examination revealed that LC progression induced thickening of alveolar walls, interstitial edema, and significant inflammatory cell infiltration. These pathological changes damaged vascular structures, exacerbated local hypoxia, and concurrently promoted angiogenesis. Critically, DBD treatment significantly delayed LC growth and reduced both local inflammation and angiogenesis, suggesting a protective role for DBD in maintaining lung tissue integrity and alleviating LC-induced damage.

### Immunohistochemical analysis of microvessel density and HIF-1α/VEGF expression

**Figure 6:**
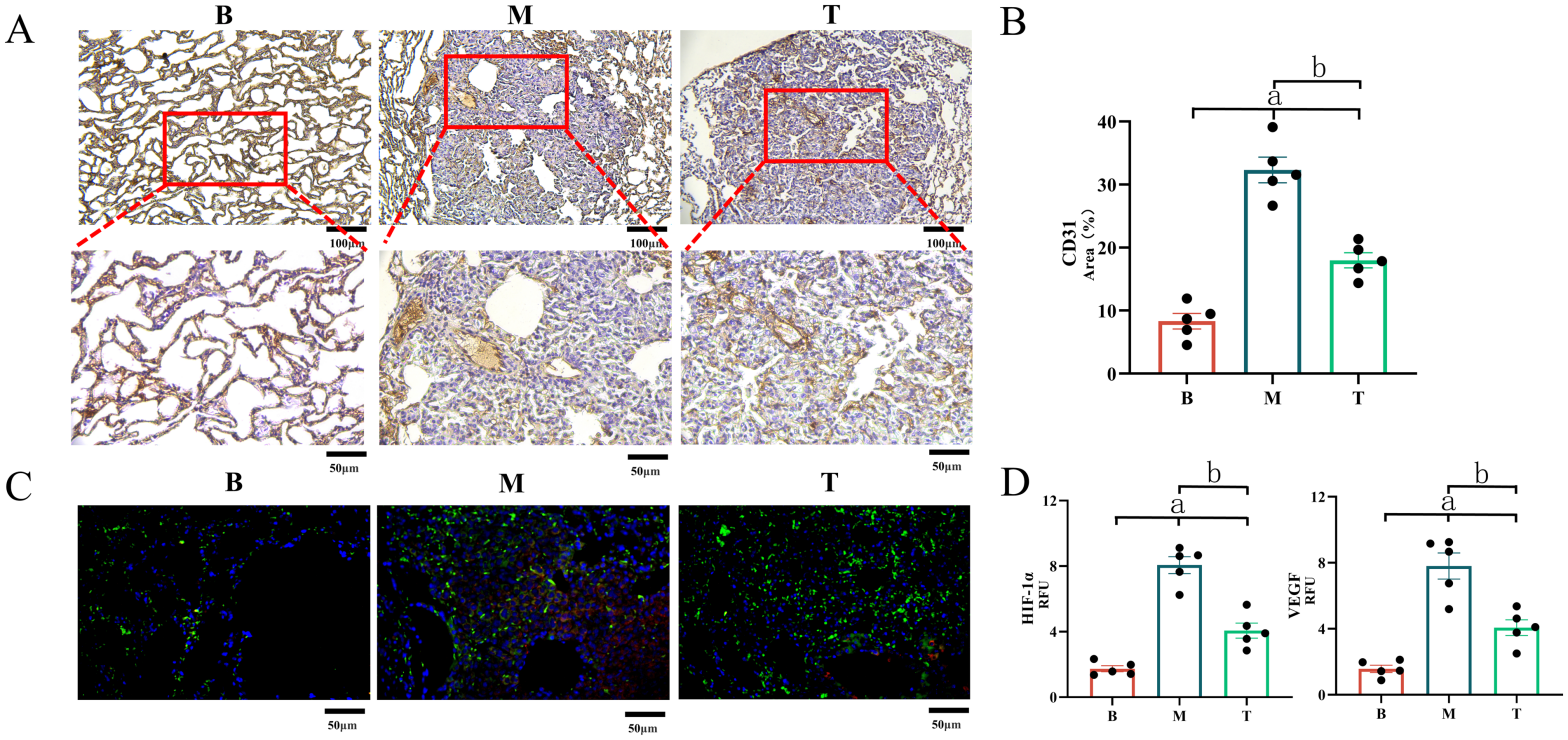
Expression of CD31, VEGF and HIF-1α in mouse lung tissue. **(A)** Demonstrates LC-induced angiogenesis, with CD31 marking local angiogenesis at the lesion site. **(B)** Demonstrates the area of CD31 in the field of view. **(C)** Depicts the expression of VEGF and HIF-1α in lung tissue observed via immunofluorescence, where red represents HIF-1α and green represents VEGF. **(D)** Represents the gray value statistics of VEGF and HIF-1α in the field of view. M denotes the model group, T denotes the DBD group, and B denotes the blank group. Statistical significance is denoted as follows: a indicates a significant difference compared with group B (*p* < 0.05), and b indicates a significant difference compared with group M (*p* < 0.05). Data are based on observations from *n* = 5 mice per group.

Since overexpression of VEGF and its upstream regulator HIF-1α plays a critical role in driving tumor angiogenesis, which provides essential nutrients for LC growth, we assessed MVD and the expression levels of HIF-1α and VEGF at lesion sites using immunohistochemistry (IHC). The positive areas of CD31 in the B, M, and T groups were 8.323 ± 1.187, 32.316 ± 1.941, and 17.995 ± 1.158%, respectively. The levels of HIF-1α were 1.738 ± 0.183, 8.060 ± 0.559, and 4.065 ± 0.488 Mean, respectively. The levels of VEGF were 1.575 ± 0.216, 7.805 ± 0.723, and 4.071 ± 0.477 RFU, respectively. LC can increase MVD by inducing the expression of HIF-1α and VEGF (B vs. M and T, *p* < 0.05). DBD treatment significantly inhibited the expression of VEGF and HIF-1α, reduced MVD (T vs. M, *p* < 0.05), and delayed the growth of LC. As shown in Figure 6A, MVD was significantly elevated in groups M and T compared to group B, with associated irregular and incomplete vascular structures. However, DBD treatment significantly reduced MVD in group T relative to group M. Furthermore, IHC analysis confirmed that DBD treatment markedly suppressed the expression of both VEGF and HIF-1αat the lesion site (Figure 6B).

### Assessment of lung function and pulmonary blood flow

**Figure 7:**
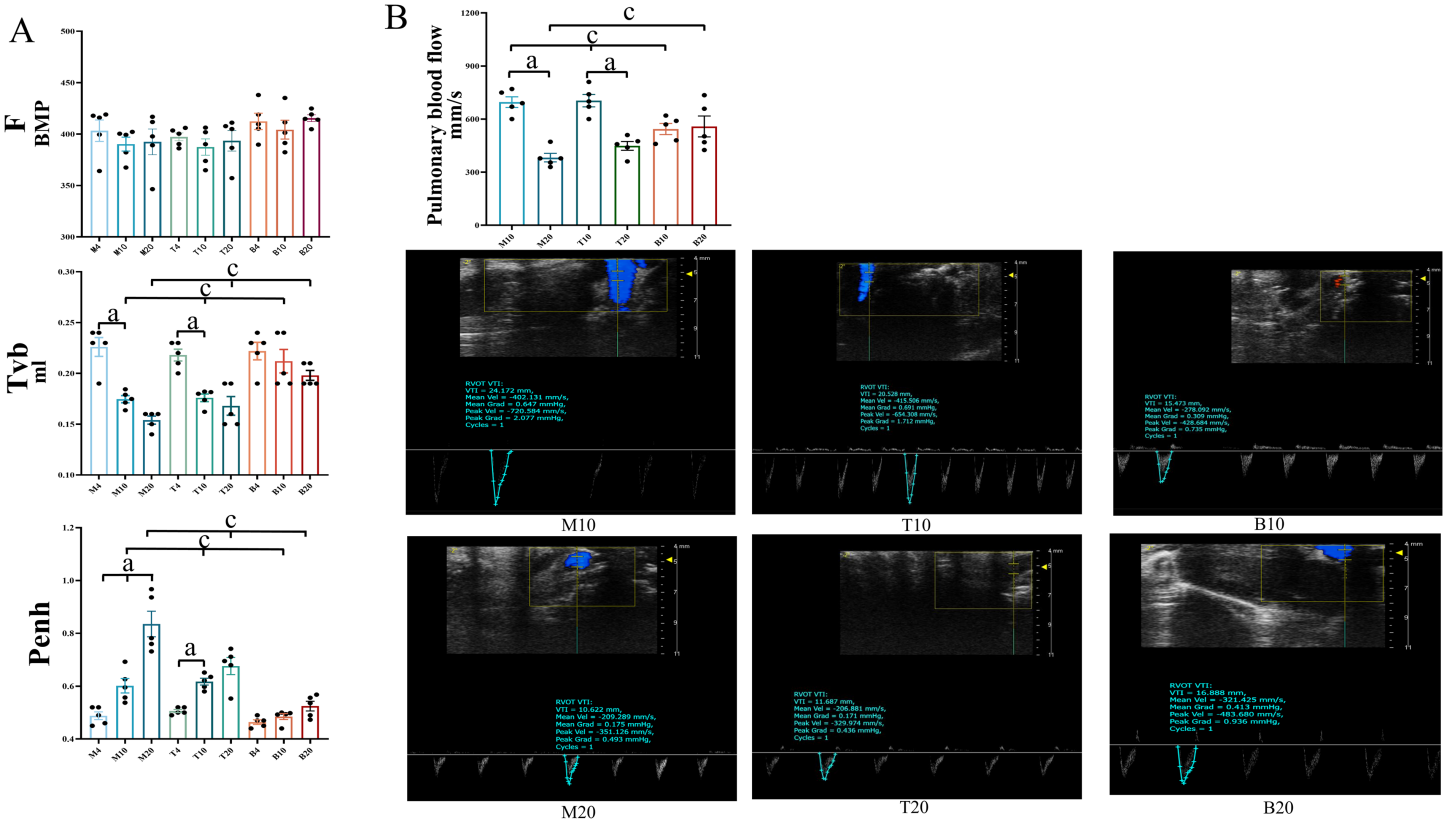
Lung function and pulmonary artery blood flow in mice. **(A)** Displays ventilation frequency (F), tidal volume (Tvb), and airway resistance (Penh) across the three groups of mice at the 4-week, 10-week, and 20-week marks, respectively. **(B)** Illustrates pulmonary artery blood flow measurements for the three groups at the 4-week, 10-week, and 20-week marks. Statistical significance is denoted as follows: a indicates a significant difference in within-group comparisons (*p* < 0.05), and c indicates a significant difference compared to group B at the same time point (p < 0.05). M represents the model group, T represents the DBD group, and B represents the blank group. M4 denotes the 4-week time point of the model group, and so forth. Data are expressed as mean ± SEM (*n* = 5).

LC progression impairs lung function, exacerbating hypoxia and consequently fueling angiogenesis. To evaluate the impact of DBD treatment on pulmonary physiology, lung function was assessed using whole-body plethysmography (WBP) at 4, 10, and 20 weeks, measuring ventilation frequency (F), tidal volume (TVb), and enhanced pause (Penh, an index of airway resistance). As shown in Figure 7A, no significant differences in lung function parameters were observed among the groups at the 4-week time point. By 10 weeks, both groups M and T exhibited a decline in F and TVb, accompanied by an increase in Penh. By 20 weeks, group M showed a further deterioration in F and TVb, with significant declines in F and TVb and increased Penh, indicating progressive lung dysfunction. In contrast, DBD-treated mice (group T) displayed improved F and TVb, along with reduced Penh compared to group M, although these differences did not reach statistical significance (*p* > 0.05). These findings suggest a trend where DBD may alleviate airway resistance and help preserve lung function over the course of LC progression. To specifically assess pulmonary hemodynamics, pulmonary artery blood flow velocity was measured using small animal ultrasound. As shown in Figure 7B that at 10 weeks, pulmonary artery blood flow velocity was significantly increased in both groups M and T compared to group B (*p* < 0.05). By 20 weeks, however, group M exhibited a significant decrease in blood flow velocity relative to group B (*p* < 0.05). Notably, DBD treatment reversed this decline, restoring blood flow velocity in group T to levels comparable to group B (*p* > 0.05).

### Assessment of hematological parameters, circulating cytokines, and liver function

**Figure 8:**
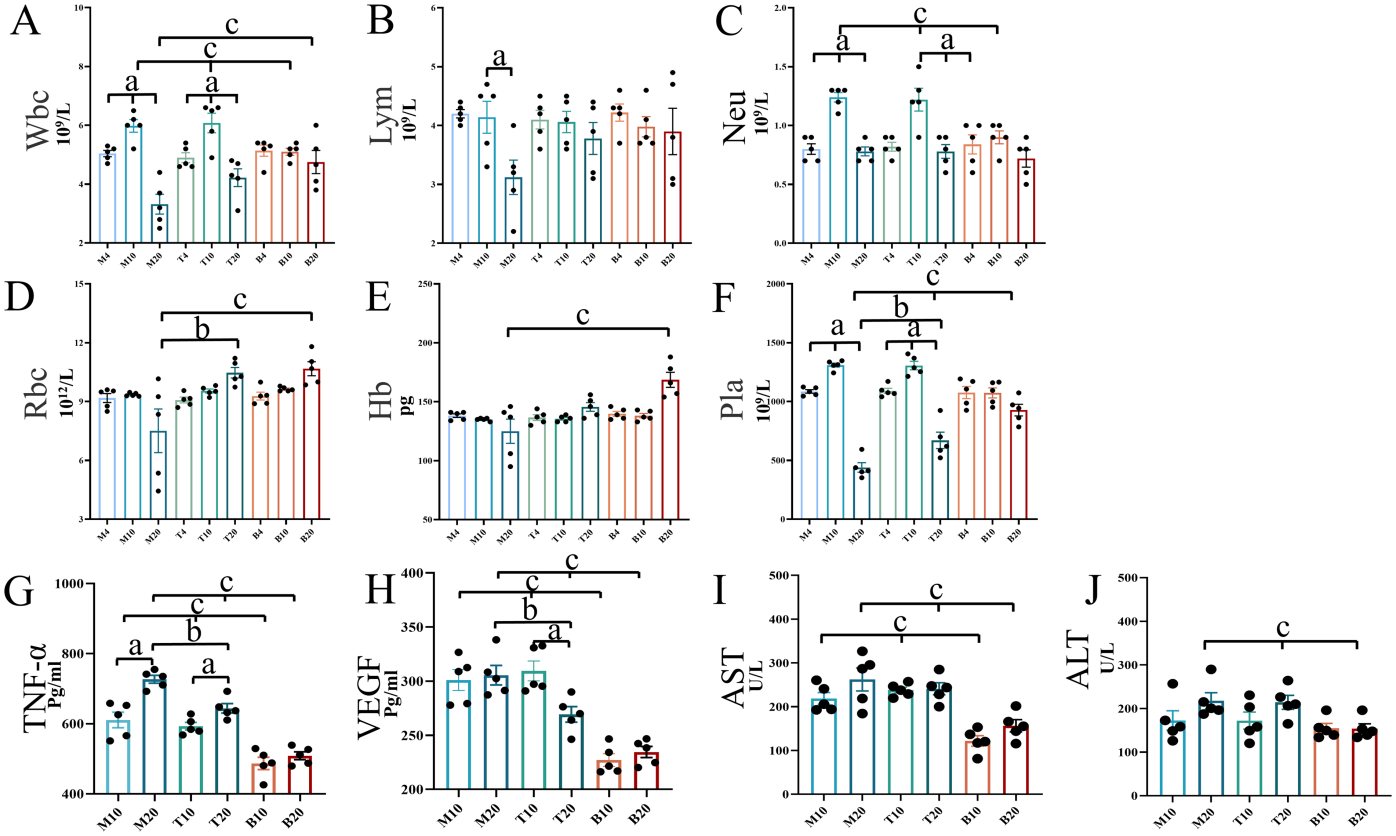
Mouse blood test results and liver function analysis. **(A–F)** Depict the concentrations of routine blood indicators in mice. **(G)** Shows the expression levels of TNF-α in mice. **(H)** Illustrates the expression levels of VEGF in mice. **(I,J)** Represent liver function indicators, AST and ALT, respectively. Statistical significance is denoted as follows: a indicates significant differences in within-group comparisons, b indicates significant differences between groups M and T at the same time point, and c indicates significant differences compared to group B at the same time point. M4 denotes the 4-week time point of the model group, and so forth. Data are expressed as mean ± SEM (*n* = 5).

Peripheral blood analysis provides critical insights into systemic responses during lung cancer (LC) progression and treatment. Blood cell counts, circulating levels of HIF-1α/VEGF/TNF-α, and liver enzyme activities were evaluated at 4, 10, and 20 weeks. As shown in Figure 8, hematological parameters and cytokine levels remained within normal ranges across all groups at the 4-week time point. By 10-week, significant alterations emerged: both the M group and the DBD T group exhibited significantly elevated levels of white blood cells (WBC), neutrophils (Neu), VEGF, and TNF-α compared to the blank control group (B) (*p* < 0.05). At the 20-week endpoint, group M displayed significant decreases in WBC, Neu, lymphocyte (LYM), and platelet (Pla) levels relative to group B, while TNF-α levels continued to rise significantly (*p* < 0.05). DBD treatment effectively delayed the decline in LYM and Pla levels, inhibited TNF-α and VEGF expression (*p* < 0.05), and increased red blood cell (RBC) and hemoglobin (Hb) concentrations (*p* < 0.05). Analysis of liver enzymes aspartate aminotransferase (AST) and alanine aminotransferase (ALT) revealed no significant increase in the DBD-treated group (T) compared to the blank control group (B) at any time point (*p* > 0.05, Figures 8I,J), indicating the absence of significant drug-induced liver burden and confirming the safety profile of DBD treatment.

## Discussion

This study integrated network pharmacology predictions with *in vivo* validation to investigate the anti-lung cancer mechanism of Danggui Buxue Decoction (DBD). Network pharmacology analysis identified angiogenesis, particularly centered around VEGFA, as a core pathway, suggesting that DBD may exert therapeutic effects by inhibiting angiogenesis (Figure 2). Subsequent experiments in an NNK-induced lung cancer model in A/J mice confirmed that DBD significantly suppressed tumor growth. Mechanistically, DBD downregulated the expression of HIF-1α and VEGF in tumor tissues (Figures 4–6), leading to reduced MVD, as quantified via CD31 immunohistochemistry (Figure 6A). These findings strongly support the conclusion that DBD delays lung cancer progression primarily through inhibition of the HIF-1α/VEGF-mediated angiogenesis axis.

The “dormant” microtumors initially rely on pre-existing vasculature for oxygen supply. However, upon “awakening,” increasing tumor mass exacerbates hypoxia, which stabilizes HIF-1α by inhibiting its proteasomal degradation (27). Elevated HIF-1α levels promote VEGF expression, a central driver of angiogenesis. Beyond VEGF, other factors including FGF, Angiopoietins, PDGF, and inflammatory cytokines such as TNF-α also contribute to angiogenic regulation (28). For instance, FGF can complement VEGF signaling, and its upregulation may lead to escape mechanisms upon VEGF inhibition (29). Ang-2 promotes vascular destabilization facilitating VEGF-induced sprouting, while PDGF enhances pericyte coverage and vessel maturation (30, 31). Inflammatory cytokines including TNF-α recruit pro-angiogenic immune cells and further upregulate VEGF, creating a vicious cycle linking hypoxia, angiogenesis and inflammation (32). Moreover, VEGF promotes immunosuppression by impairing dendritic cell maturation and T-cell function, and facilitates metastasis via induction of epithelial-mesenchymal transition (EMT) (33, 34). These multifaceted roles of VEGF underscore why it remains a key therapeutic target. Current anti-angiogenic agents include monoclonal antibodies (e.g., ramucirumab), small-molecule kinase inhibitors (e.g., nintedanib, anlotinib, lenvatinib), and dual-targeting agents such as bispecific antibodies against PD-1/VEGF (e.g., PM8002) or DLL4/VEGF (e.g., ES104) (35, 36). Notably, our network pharmacology analysis also suggested involvement of the TNF-α and AKT pathways (Figure 2), implying that DBD may act through a multi-target mechanism.

Since its production in 1971, anti-angiogenic therapy has become an important strategy of lung cancer treatment. However, monotherapy often faces limitations including adverse effects (e.g., hypertension, proteinuria), exacerbated hypoxia, and compensatory activation of alternative pathways (e.g., FGF, Ang-2), leading to drug resistance (37). Combination strategies with immunotherapy or chemotherapy are thus recommended, though challenges regarding toxicity and response heterogeneity remain. Natural products like DBD offer a promising alternative due to their multi-component nature and favorable safety profiles (38). In this study, DBD not only reduced VEGF and TNF-α levels but also improved overall physiological conditions—enhancing immune function (increased lymphocytes), supporting hematopoiesis (stable RBC and hemoglobin), regulating coagulation (platelet stabilization), and ameliorating pulmonary hypoxia (improved arterial blood flow and lung function). This systemic improvement helps disrupt the “hypoxia-angiogenesis-inflammation” cycle, thereby suppressing tumor progression. The NNK-induced A/J mouse model is a well-established model resembling human smoking-associated lung adenocarcinoma, characterized by Kras mutations and chronic inflammation (39). Compared to other models, it offers high reproducibility, clinical relevance, and lower mortality (40). However, it does not fully recapitulate human tumor heterogeneity and progression. Future studies should validate DBD in more advanced models, such as patient-derived xenografts or genetically engineered mice, and explore its combinatory potential with chemo-, targeted, or immunotherapies (41).

DBD is a classic prescription in traditional Chinese medicine for tonifying Qi and generating blood. Its therapeutic effects on LC are manifested in multiple aspects. In TCM theory, “Qi” represents vital energy and defensive function, which is closely associated with immune competence, while the effect of “activating blood circulation” is traditionally described as resolving stasis and promoting normal circulation (42, 43). Our results demonstrated that DBD treatment significantly increased the counts of immune cells and oxygen-carrying cells in the blood and improved pulmonary blood oxygen supply, which reflects the TCM action of “tonifying Qi and generating blood.” The “Qi-tonifying” effect improves oxygen supply—as evidenced by increased hemoglobin levels and enhanced pulmonary function—thereby alleviating hypoxia (44). This reduction in hypoxia leads to decreased stabilization of HIF-1α, resulting in the downregulation of VEGF expression and inhibition of pathological angiogenesis (“blood activation”). Meanwhile, the mitigation of hypoxia and reduction in VEGF help alleviate immunosuppression, further enhancing “Qi”-related immune functions. In addition, the anti-angiogenic effect (“blood activation”) also improves blood perfusion and reduces inflammation, which in turn supports immune cell function and reduces hypoxic stress (“Qi tonification”) (45, 46). Studies have indicated that different ratios of AR and ARS can lead to variations in the release of active pharmaceutical ingredients, vasodilatory capacity, and therapeutic outcomes, which may underlie the conceptual importance of herbal compatibility in TCM (16, 47, 48).

This study has several limitations. First, the sample size (*n* = 5 per group), though consistent with common practice, may limit statistical power and generalizability (49, 50). Second, while we demonstrated DBD’s inhibition of the HIF-1α/VEGF axis, the specific bioactive compounds within DBD and their precise targets remain unclear. Third, although DBD alleviated immunosuppression, its effects on specific immune subsets (e.g., T cells, NK cells, MDSCs, Tregs) and their functional states require further elucidation (51). Future work should integrate phytochemistry, pharmacokinetics, and functional genetics to identify active components and their mechanisms, alongside detailed immune profiling to fully unravel the scientific basis of DBD’s “Yiqi Shengxue” effects (52).

## Conclusion

In summary, this study establishes that DBD effectively delays the progression of NNK-induced lung cancer in a clinically relevant A/J mouse model. Its core mechanism involves disrupting the hypoxia-driven HIF-1α/VEGF signaling axis, thereby inhibiting pathological angiogenesis. DBD exerts multi-faceted protective effects: preserving lung tissue integrity, improving pulmonary perfusion and mitigating airway resistance (alleviating hypoxia), suppressing local and systemic inflammation (notably TNF-α), and modulating systemic immune/hematopoietic function (maintaining lymphocytes, red blood cells, and platelets). Importantly, DBD achieves these therapeutic benefits without inducing detectable hepatotoxicity, highlighting its potential as a safe and effective complementary therapeutic strategy, particularly for managing angiogenesis and inflammation in smoking-associated lung cancer. Future studies should focus on identifying the specific active compounds within DBD responsible for these effects and further elucidating the detailed molecular pathways involved in its anti-angiogenic and immunomodulatory actions.

## Data Availability

The original contributions presented in the study are included in the article/Supplementary material, further inquiries can be directed to the corresponding authors.

## Footnotes

^1^ https://www.cacm.org.cn/2024/08/02/29432/

^2^ https://www.uniprot.org/

^3^ https://www.genecards.org/

## Glossary

ALT: Alanine transaminase
ARS: *Angelica sinensis* (Oliv.) Diels (dang gui)
AR: *Astragalus membranaceus* (Fisch.) Bge. var. mongholicus (Bge.) Hsiao (Huang Qi)
AST: Aspartate transaminase
B: Blank group
CT: Computed tomography
DBD: Danggui Buxue Decoction
ELISA: Enzyme-linked immunosorbent assay
Hb: Hemoglobin
HIF-1α: Hypoxia-inducible factor-1α
LC: Lung cancer
M: Model group
Pla: Platelet
Neu: Neutrophil
F: Respiratory rate
TVb: Tidal volume
RBC: Red blood cell
TCM: Traditional Chinese Medicine
T: Treatment group
TNF-α: Tumor necrosis factor-α
VEGF: Vascular endothelial growth factor
WBP: Whole body plethysmography
WBC: White blood cell
NNK: 4-(methylnitrosamino)-1-(3-pyridyl)-1-butanone
