# Supplementary material for: Danggui Buxue Decoction attenuates 4-(methylnitrosamino)-1-(3-pyridyl)-1-butanone—induced lung cancer growth in A/J mice by suppressing HIF-1α/VEGF-mediated angiogenesis
Source: Front Med (Lausanne). 2025 Oct 22;12:1687685. doi: 10.3389/fmed.2025.1687685 (PMC12589919; doi:10.3389/fmed.2025.1687685)
Supplement: Supplementary file 1 [file Table_1.DOCX]

| Number | Degree | name |
| --- | --- | --- |
| 1 | 54 | AKT1 |
| 2 | 53 | TP53 |
| 3 | 52 | VEGFA |
| 4 | 51 | TNF |
| 5 | 49 | CASP3 |
| 6 | 49 | HIF1A |
| 7 | 49 | EGFR |
| 8 | 49 | IL6 |
| 9 | 48 | MYC |
| 10 | 47 | CCND1 |
| 11 | 47 | IL1B |
| 12 | 47 | PTGS2 |
| 13 | 47 | MMP9 |
| 14 | 43 | ERBB2 |
| 15 | 43 | MMP2 |
| 16 | 43 | PPARG |
| 17 | 43 | CXCL8 |
| 18 | 40 | BCL2L1 |
| 19 | 39 | IL10 |
| 20 | 38 | CASP8 |
| 21 | 37 | KDR |
| 22 | 37 | TGFB1 |
| 23 | 36 | MAPK1 |
| 24 | 36 | ICAM1 |
| 25 | 36 | CDKN2A |
| 26 | 35 | MAPK14 |
| 27 | 34 | IL2 |
| 28 | 34 | STAT1 |
| 29 | 33 | NOS3 |
| 30 | 33 | CASP9 |
| 31 | 32 | GSK3B |
| 32 | 32 | SPP1 |
| 33 | 32 | MAPK8 |
| 34 | 31 | AR |
| 35 | 31 | IFNG |
| 36 | 31 | VCAM1 |
| 37 | 31 | CAV1 |
